# Efficient Bayesian inference of instantaneous reproduction numbers at fine spatial scales, with an application to mapping and nowcasting the Covid‐19 epidemic in British local authorities

**DOI:** 10.1111/rssa.12971

**Published:** 2022-12-08

**Authors:** Yee Whye Teh, Bryn Elesedy, Bobby He, Michael Hutchinson, Sheheryar Zaidi, Avishkar Bhoopchand, Ulrich Paquet, Nenad Tomasev, Jonathan Read, Peter J. Diggle

**Affiliations:** ^1^ Department of Statistics University of Oxford Oxford UK; ^2^ Department of Statistics University of Oxford, seconded from DeepMind Oxford UK; ^3^ CHICAS, Lancaster Medical School Lancaster University Lancaster UK

## 1 INTRODUCTION

The spatio‐temporal pattern of Covid‐19 infections, as for most infectious disease epidemics, is highly heterogenous as a consequence of local variations in risk factors and exposures. Consequently, the widely quoted national‐level estimates of reproduction numbers are of limited value in guiding local interventions and monitoring their effectiveness. It is crucial for national and local policy‐makers, and for health protection teams, that accurate, well‐calibrated and timely predictions of Covid‐19 incidences and transmission rates are available at fine spatial scales. Obtaining such estimates is challenging, not least due to the prevalence of asymptomatic Covid‐19 transmissions, as well as difficulties of obtaining high‐resolution and high‐frequency data. In addition, low case counts at a local level further confounds the inference for Covid‐19 transmission rates, adding unwelcome uncertainty.

In this paper we develop a hierarchical Bayesian method for inference of transmission rates at fine spatial scales. Our model incorporates both temporal and spatial dependencies of local transmission rates in order to share statistical strength and reduce uncertainty. It also incorporates information about population flows to model potential transmissions across local areas. A simple approach to posterior simulation quickly becomes computationally infeasible, which is problematic if the system is required to provide timely predictions. We describe how to make posterior simulation for the model efficient, so that we are able to provide daily updates on epidemic developments.

The results can be found at our web site https://localcovid.info, which is updated daily to display estimated instantaneous reproduction numbers and predicted case counts for the next weeks, across local authorities in Great Britain. The codebase updating the web site can be found at https://github.com/oxcsml/Rmap. We hope that our methodology and web site will be of interest to researchers, policy‐makers and the public alike, to help identify upcoming local outbreaks and to aid in the containment of Covid‐19 through both public health measures and personal decisions taken by the general public.

## 2 DATA

Our model is applied to publicly available daily counts of positive test results reported under the combined Pillars 1 (NHS and PHE) and 2 (commercial partners) of the UK's Covid‐19 testing strategy.^1^ The data are available for 312 lower‐tier local authorities (LTLAs) in England, 14 NHS Health Boards in Scotland (each covering multiple local authorities) and 22 unitary local authorities in Wales, for a total of n=348 local areas. The data are daily counts of lab‐confirmed (PCR swab) cases presented by specimen date, starting from 30 January 2020. The original data are from the respective national public health authorities of England^2^, Scotland^3^and Wales^4^and we access them through the DELVE Global Covid‐19 Dataset^5^ (Bhoopchand et al., 2020). Due to delays in processing tests, we ignore the last 7 days of case counts.

## 3 METHOD

Our method is based on an approach to infectious disease modelling using discrete renewal processes. These have a long history, and have served as the basis for a number of recent studies estimating instantaneous reproduction numbers, (Cori et al., 2013; Flaxman et al., 2020; Fraser, 2007; Wallinga & Teunis, 2004). See Bhatt et al. (2020) and references therein for historical and mathematical background, as well as Gostic et al. (2020) for important practical considerations.

Following Flaxman et al. (2020), we model latent time series of incidence rates via renewal processes, and separate observations of reported cases using negative binomial distributions, to account for uncertainties in case reporting, outliers in case counts, and delays between infection and testing. We introduce a number of extensions and differences addressing issues that arise for applications to modelling epidemics at local authority level rather than regional or national levels. Firstly, we introduce dependencies between reproduction numbers across neighbouring localities, in order to smooth estimates of reproduction numbers and statistical strength across localities and time. We do this using a spatio‐temporal Gaussian process (GP) prior for the log‐transformed reproduction numbers. Secondly, we model transmissions across localities using a spatial meta‐population model. Our meta‐population model incorporates commuter flow data from the UK 2011 Census in order to capture stable patterns of heterogenous cross‐infection rates among local authorities, linked to typical commuter patterns. Human mobility patterns may also reflect the introduction of non‐pharmaceutical interventions (NPIs), though our model does not explicitly use real‐time mobility data so cannot estimate the direct or indirect effects of NPIs.

The model is implemented in the Stan probabilistic programming language (Carpenter et al., 2017), which uses the No‐U‐Turn Sampler (NUTS) (Hoffman & Gelman, 2014) for posterior simulation. A number of modelling design choices as well as inference approximations are made to improving mixing and computational efficiency. These are described in Appendix B.

### 3.1 Model overview

In this section we give an overview of our model, which we refer to as EpiMap. The model consists of three layers: a latent Gaussian process over the log reproduction numbers, a meta‐population model for the epidemics across local areas and an observation model relating the size of the epidemic with the observed number of positive tests in each day and area.

We first introduce some notations. We are interested in estimating the instantaneous reproduction numbers, $R_{i,t}$, across local areas in the United Kingdom (indexed by i) and across time (indexed by t). For each local area i and day t, the observed daily Pillars 1 + 2 case counts are denoted $C_{i,t}$. Let the unobserved daily infection (incidence) counts be $X_{i,t}$.

Starting with the observation model, we model the number of reported cases using a delay distribution and an over‐dispersed negative binomial observation model:

(1) $$C_{i,t}|X_{i,1:t},\phi_{i}∼\text{NegBin}(V_{\text{day\_of\_week}(t)}E_{i,t},\phi_{i}),\;E_{i,t}=\overset{t}{\underset{s=1}{\sum }}\;X_{i,t-s}D_{s},$$

where $D_{s}$ is the probability that an infected person gets tested and tests positive s days after infection and $E_{i,t}$ is the expected number of positive test cases on day t in area i. NegBin(μ,ϕ) is the negative binomial distribution with mean μ and dispersion parameter ϕ, while $V_{\text{day\_of\_week}(t)}$ models day‐of‐week variations in reported cases. Section 3.1.2 gives more details.

Assuming a homogeneously mixing population in each area, and interactions across areas modelled using a cross‐coupled meta‐population model, we model the number of new infections in each area as follows. Conditional on the history of infections, let

(2) $$Z_{i,t}=\overset{t}{\underset{s=1}{\sum }}\;X_{i,t-s}W_{s},$$

be the infection load on day t caused by previous infections in area i, if each primary case produces one secondary case. $W_{s}$ describes the generation distribution, and is the probability that a secondary infection occurs s days after the primary infection. See Section 3.1.2 for more details on how we parameterise $W_{s}$. These secondary infections can occur in area i, or in another area, for example due to individuals working in an area different from where they live. We model this with a time‐dependent flux matrix $F_{ji}^{\left(t\right)}$, which is interpreted as the probability that a primary case living in area j infects a secondary case living in area i on day t. The resulting cross‐coupled infection load in area i is:

(3) $${\tilde{Z}}_{i,t}=\overset{n}{\underset{j=1}{\sum }}\;F_{ji}^{\left(t\right)}Z_{j,t}.$$

We describe the meta‐population model in further detail in Section 3.1.3, including how the flux matrices are parameterised. We model the number of new infections on day t as,

(4) $$X_{i,t}|R_{i,t},X_{1:n,1:t-1}∼\text{NegBin}(R_{i,t}{\tilde{Z}}_{i,t},\psi ),$$

where $R_{i,t}{\tilde{Z}}_{i,t}$ is the force of infection in area i and day t, and ψ is a dispersion parameter which allows for over‐dispersion. We expect this to be a better model for Covid‐19 than using a Poisson distribution in (4) due to super‐spreading events. Note that if we used a Poisson then the secondary infections resulting from a primary infection would have been modelled as conditionally iid given the primary infection. The use of a negative binomial distribution instead introduces a positive correlation among the secondary infections.

In order to make the posterior simulation computationally efficient using Stan, we approximated this with a positivised Gaussian distribution; see Appendix B.2.

#### 3.1.1 Latent GP

With low case counts, inferring $R_{i,t}$ over small local areas can lead to high uncertainty. A standard Bayesian hierarchical modelling approach is to borrow strength across different local areas and across different time points. We use GPs to do so; namely, for area i and time t we model:

(5) $$R_{i,t}=\exp(S_{i,t}+U_{i,t}),$$

where $S_{:,:}$ is a GP with a separable Matern(1/2) kernel^6^:

(6) $$\;\text{Cov}(S_{i,s},S_{j,t})={\left(\sigma^{\text{spatial}}\right)}^{2}\exp(-\|y_{i}-y_{j}\|/\rho^{\text{spatial}}-\|s-t\|/\rho^{\text{time}}),$$

and $U_{i,:}$ are independent copies of a GP with Matern(1/2) kernels:

(7) $$\;\text{Cov}(U_{i,s},U_{i,t})={\left(\sigma^{\text{local}}\right)}^{2}\exp(-\|s-t\|/\rho^{\text{time}}).$$

Here, $y_{i}$ and $y_{j}$ are the geographical centres of areas i and j, respectively, s and t are daily indices for each Monday, and we assume that the instantaneous reproduction numbers are constant within each week (taken to be Monday to Sunday). Note that our prior covariances in Equations (6) and (7) enjoy a Kronecker structure across the space and time dimensions, which allows for efficient computations (see Section B.1). In the temporal case, which is one‐dimensional, the GP prior with the Matern(1/2) kernel is equivalent to an AR(1) process with zero mean. We also considered Matern(3/2), Matern(5/2) and squared‐exponential covariance kernels, which produced similar inferences.

The hyperparameters of the spatio‐temporal GP are: scale parameters $\sigma^{\text{spatial}}$ and $\sigma^{\text{local}}$ and length‐scale parameters $\rho^{\text{spatial}}$ and $\rho^{\text{time}}$. We place independent truncated normal priors ${𝒩}_{+}(0,0.5)$ over the scale parameters. For the length scale parameters, we have found that if we inferred these along with the rest of the random variables in the model, the posterior distribution places mass on large spatial length scales and short temporal length scales. This has an undesirable over‐generalisation effect, and we believe this behaviour is due to model misspecification with respect to the length scale parameters. Instead we selected these using an initial cross validation run optimising for performance of forecasted case counts three weeks into the future, and selected $\rho^{\text{spatial}}=10\;km$ and $\rho^{\text{temporal}}=200$ days.

#### 3.1.2 Observation and infection model

Weekly variations are modelled using multiplicative factors in (1), with a uniform prior over positive vectors of length 7 and sums to 7. Following Flaxman et al. (2020) we use an over‐dispersed negative binomial observation model (1), with a broad half normal prior for the dispersion parameters, $\phi_{i}∼{𝒩}_{+}(0,5)$ iid. The neg_binomial_2 parameterisation in Stan uses a mean parameter μ, an inverse‐dispersion parameter c, and variance $\mu +\mu^{2}/c$. We use a different parameterisation, and set c=μ/ϕ, where ϕ is a dispersion parameter. This gives a variance of (1+ϕ)μ and probability mass function:

(8) $$p(x|\mu ,\phi )=\left(\begin{array}{c} x+\mu /\phi -1\\ x \end{array}\right){\left(\frac{\phi }{1+\phi }\right)}^{x}{\left(\frac{1}{1+\phi }\right)}^{\mu /\phi }.$$

This parameterisation naturally emphasises the infinite divisibility of the negative binomial, that is, if $Y_{1},\ldots ,Y_{m}$ are independent negative binomial random variables with means $\mu_{1},\ldots ,\mu_{m}$ and the same dispersion parameter ϕ, then $\sum_{i=1}^{m}Y_{i}$ is also negative binomially distributed with mean $\sum_{i=1}^{m}\mu_{i}$ and dispersion ϕ, a sensible choice in cases where we believe counts are sums of independent random events.

The infection‐to‐test delay distribution $D_{s}$ is a convolution of two delay distributions: an incubation period distribution, and a symptom‐onset‐to‐test distribution. Following Bi et al. (2020), we use a $\text{LogNormal}(\mu ,\sigma^{2})$ distribution for the incubation period, where μ has a 95% confidence interval (CI) of (1.44, 1.69) and mode 1.57, and $\sigma^{2}$ has 95% CI of (0.56, 0.75) with mode 0.65. This results in a median of 4.8 days and a 90% confidence interval of (1.64,14.04) days for the incubation period, and we assume an additional 2‐day delay to get tested.

Similarly, we parameterise the generation distribution $W_{s}$ as a Gamma distribution whose shape parameter has mode 2.29 with (1.77, 3.34) 95% CI, and whose rate parameter has mode 0.36 with (0.26, 0.57) 95% CI. This corresponds to the serial interval parameter distributions from Bi et al. (2020); we note that the serial interval is often used as an accessible proxy for the unobserved generation distribution (Cori et al., 2013). For both $D_{s}$ and $W_{s}$, we aggregate predictions and inferences from 10 bootstrapped runs of our model, each with independently sampled LogNormal and Gamma parameters respectively. This is equivalent to a nested Monte Carlo approximation to a cut or modular model (Carmona & Nicholls, 2020; Jacob et al., 2017; Plummer, 2015). We found this to be crucial to avoiding overconfident predictions for $R_{t}$ estimates.

For the dispersion paramter ψ, we use a weakly informative prior $\psi ∼{𝒩}_{+}(0,2.5)$.

#### 3.1.3 Meta‐population model

Our final extension relaxes the assumption in many infectious disease models, that the epidemic is evolving in a homogeneously mixing population in an area, with no significant transmissions from other areas. While this might be sensible in large regions or countries, it is not a sensible assumption for modelling multiple small areas with likely a significant number of cross‐area transmissions. To address these transmissions, we describe a simple cross‐coupled meta‐population extension, given by Equations (3) and (4).

In the following we describe how to parameterise the flux $F_{ji}$, which describes the chance that, if a primary case living in area j infects a secondary case, the secondary case will live in area i. One sensible choice, if the data were available, would be to use real‐time data on the actual volume of travel between each pair of areas. Such data are unfortunately not publicly available, and in any case the relationship between the volume of travel and the number of transmissions is not straightforward due to heterogeneity in the population.

We use commuting flow data from the 2011 Census^7^to parameterise a weekly varying flux matrix. First, the data give, after some preprocessing, a matrix M such that for each pair of areas i and j the number of individuals who live in area j and commute to work in area i is $M_{ji}$. Let $P_{j}$ be the population of area j. We take $M_{jj}$ to be the population who commute within their own area or who do not commute, so $\sum_{i}M_{ji}=P_{j}$. We consider three types of transmissions: an individual living in area j infecting another individual in area j (e.g. household transmissions), an individual living in area j working in area i infecting one living in area i, and an individual living in area i being infected while working in area j. These three types of transmissions can be described using three flux matrices:

(9) $$F_{ji}^{\text{id}}=\delta_{ji}\;F_{ji}^{\text{fwd}}=\frac{M_{ji}}{\sum_{k}M_{jk}}\;F_{ji}^{\text{rev}}=\frac{M_{ij}}{\sum_{k}M_{kj}},$$

where $\delta_{ji}=1$ if j=i and 0 otherwise. Then, we parameterise the overall flux matrix during week t using a convex combination of $F^{\text{id}}$, $F^{\text{fwd}}$, and $F^{\text{rev}}$,

(10) $$F^{\left(t\right)}=\alpha_{t}F^{\text{id}}+(1-\alpha_{t})(\beta F^{\text{fwd}}+(1-\beta )F^{\text{rev}}),$$

with $\alpha_{t}\in (0,1)$ governing the amount of mixing across areas on week t (roughly the proportion of the population working from home), and β∈(0,1) governing the amount of home‐to‐work versus work‐to‐home transmissions. We use a uniform prior over β and a weekly AR(1) prior for the log‐odds, specifically $\alpha_{t}=1/(1+\exp(-\mu_{\alpha }+\sigma_{\alpha }A_{t}))$ where the AR(1) process is given by $A_{1}∼𝒩(0,1)$, $A_{t}|A_{t-1}∼𝒩(\delta_{\alpha }A_{t-1},\sqrt{1-\delta_{\alpha }^{2}})$, with weakly informative hyperpriors $\mu_{\alpha }∼𝒩(0,0.5)$, $\sigma_{\alpha }∼{𝒩}_{+}(0,0.5)$, while the hyperprior $\delta_{\alpha }∼{𝒩}_{\left[0,1\right]}(1,1-e^{-0.25})$ is a weakly informative prior on the time scale of the AR(1) process centred around 4 weeks.

## 4 EMPIRICAL EVALUATIONS

In this section, we report some empirical evaluations of our model, which we call EpiMap. We compared two variants of EpiMap: one which models each local area separately from the rest (hence no meta‐population model nor spatial component of GP), and the other the full model. For the full model we have found that the inferences are sensitive to the length scale of the spatial GP, and so we compared the full model with varying spatial length scales and with no spatial GP component. To account for uncertainty in the serial interval and incubation period distributions, we ran EpiEstim with 10 instantiations of these distributions with parameters drawn iid from the posterior distributions reported in Bi et al. (2020), and averaged the posterior predictive distributions over these. This procedure can be interpreted as nested Monte Carlo for a cut distribution where we specified the prior for these parameters but disallow the model from updating the prior to a posterior (Plummer, 2015). We also compared against EpiEstim (Cori et al., 2013) and EpiNow2 (Abbott et al., 2020). We compared these methods on simulated data and on predicting future case counts in British local authorities. We also report estimates of $R_{t}$ at regional and national levels.

### 4.1 Simulation data

One sanity check of our method is to fit the models to simulated data for which we know the underlying $R_{t}$, and check how well our models can recover this. In this section we do just this, and compare the results with a number of other common methods.

The simulation model we use is exactly the generative model we described. We use the median distribution parameters given by Bi et al. (2020) for the serial interval and incubation period. We assume the delay distribution is the incubation period distribution plus a fixed reporting delay of 2 days.

**FIGURE 1:**
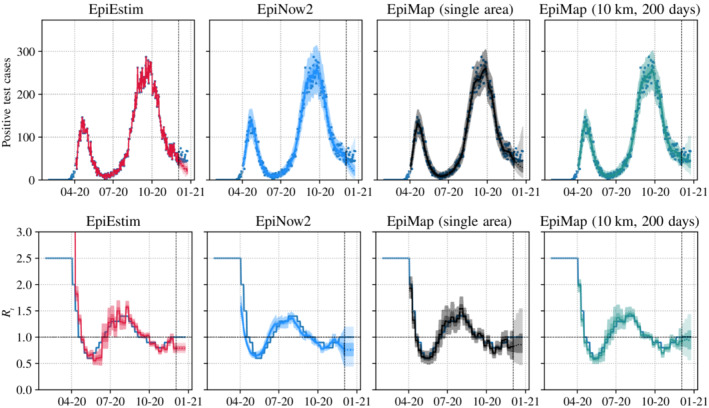
Top: Estimated median, 50% (inner) and 95% (outer) credible intervals of the posterior predictive distributions for $C_{i,t}$, along with observed case counts. Credible intervals to the right of the vertical line are future predictions. For EpiMap, we include the day‐of‐week variation in expected cases in the model, but plot the predictive distributions without this variation for clarity. EpiEstim does not model dependence of $R_{t}$ over time, so we used the last inferred $R_{t}$ distribution for future predictions. Bottom: Estimated median, 50% (inner) and 95% (outer) credible intervals of $R_{t}$ for the methods, along with the true $R_{t}$ (piecewise constant blue line) used to create the simulated epidemic. Plots shown only for Oxford, those for the four surrounding local authorities are given in Section C.1. EpiEstim estimates are more variable because of the higher variation in observed case counts $C_{i,t}$ as compared to the latent infection counts $X_{i,t}$, and because it does not model dependence of $R_{t}$ over time. We used the last inferred $R_{t}$ distribution for future predictions for EpiEstim. [Colour figure can be viewed at wileyonlinelibrary.com]

The data are simulated by taking initial real case data from Oxford and the four surrounding LTLAs up to 14 March 2020, and from that point simulating new cases using the model. The main unspecified parameter is the $R_{t}$ in each region over time. An $R_{t}$ curve was manually designed in order to give a double peak epidemic similar in nature to the pattern seen across the United Kingdom, with case numbers in the regions roughly similar. The same $R_{t}$ curve was shared across the LTLAs. Additionally we use 50:50 flux proportions of the forward and reverse commuter flow data, with a constant $\alpha_{t}$ of 0.45. These choices of parameters are somewhat arbitrary and were chosen to give qualitatively sensible epidemic curves. To these simulated data we fit the two variations of our model, with the full model using a temporal length scale of 200 days and a range of spatial length scales between 1 and 100 km. The results can be seen in Figure 1. Plots showing the full sweep of spatial length scales for EpiMap can be found in Section C.1.

### 4.2 Predicting future case counts

Next, we evaluate the methods' predictions of future case counts by comparing them to true case counts. In addition to measuring predictive performance, we also assess the model's uncertainty calibration by comparing the coverage probability of its prediction intervals with the actual, achieved (empirical) coverage. We first picked four well‐separated dates: 12 October 2020, 23 November 2020, 21 December 2020 and 18 January 2021. For each date, we used the 15 preceding weeks of data for inference and evaluated predictions of case counts for the subsequent 3 weeks. These assessment periods were chosen to cover a range of situations from relatively stable transmission rates (during lockdown in January) to drastic changes in transmission rates due to NPIs (during December period). Note that since the methods do not model drastic changes arising from NPIs changing, we expect them to perform poorly during such periods. In addition to the variants of EpiMap, EpiEstim and EpiNow2, we also included two simple baselines: ‘zero’ which predicts zero cases for all dates and LTLAs, and ‘last case count’ which predicts using the case count on the last day of the 15‐week inference period for each LTLA.

**FIGURE 2:**
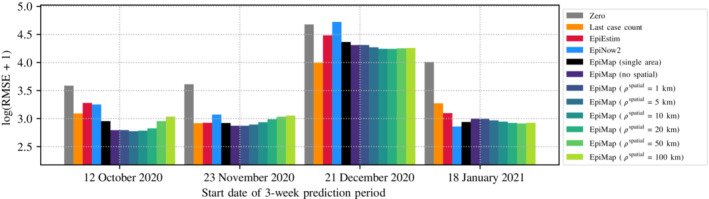
A comparison of models for predicting future case counts over a 3‐week period. Each model is fitted on 15 weeks of data and makes predictions for the following 3 weeks. Zero means predicting counts of 0, while Last case count means using the case count on the last available date for predictions. [Colour figure can be viewed at wileyonlinelibrary.com]

Figure 2 shows log(RMSE+1) between predicted and true case counts. More precisely, the RMSE is separately computed for each LTLA's predictions over the test period, then we average the resulting log(RMSE+1) across LTLAs. The log transformation is so that results are not dominated by areas with much higher case counts. EpiMap variants usually perform the best or competitively at predicting the true case counts. The positive impact of modelling cross‐area dependencies is observed, since EpiMap (single area) tends to slightly underperform the other variants of EpiMap. Moreover, the predictive performance of EpiMap is dependent on, though not very sensitive to, the choice of $\rho^{\text{spatial}}$. Note that for the start date 21 December 2020, all models perform worse relative to other dates. This is because of significant changes in the dynamics of Covid‐19 spread due to changing NPIs over the Christmas period, information that is not incorporated into any of these models.

**FIGURE 3:**
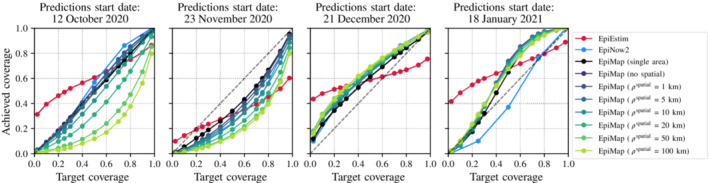
Reliability curves assessing the uncertainty estimates produced by models. Each model yields percentiles of its case count posterior predictive distribution. The curves show the portion $\hat{p}$ of predictions (across dates and lower‐tier local authorities) for which the true case count is less than the *p*th percentile ${ĉ}_{p}$ of the model (y‐axis) versus p (x‐axis). [Colour figure can be viewed at wileyonlinelibrary.com]

Figure 3 assesses the quality of the uncertainty estimates produced by the models using reliability curves. Each model outputs percentiles of the posterior predictive distribution of case counts. Let ${ĉ}_{p}$ be the *p*th percentile produced by a model for a given date and LTLA. Ideally, we expect that the percentage of dates and LTLAs for which the true case count c is less than or equal to ${ĉ}_{p}$, is approximately p. In other words, the actual, empirical coverage of the *p*th percentile (y‐axis of Figure 3) will ideally be equal to the target coverage p (x‐axis of Figure 3), yielding a reliability curve close to y=x. We observe that EpiMap's uncertainty estimates generally capture the underlying case counts distribution well, though with some variation across start dates and model configurations. EpiNow2 usually performs similar to the well‐performing configurations of EpiMap. EpiEstim's uncertainty estimates are overconfident as indicated by the flatter‐shaped curves. For the first three start dates, EpiMap (single area) and models with small $\rho^{\text{spatial}}$ yield better uncertainty estimates. For 21 December 2020, the concave shape of the reliability curves indicates that models are overestimating case counts, which is consistent with the fact that stricter NPIs curbed case counts while the models predicted case counts would increase assuming no changes in spread dynamics. For 18 January 2021, larger $\rho^{\text{spatial}}$ perform best, likely because the prevailing *national* lockdown in that period meant that spread dynamics were more uniform across areas. Additional results are in Appendix C.2, including loss and reliability curves stratified by week during the 3‐week prediction period and individual LTLA losses.

### 4.3 Regional estimates

While our model operates at the level of local authorities, we can estimate $R_{t}$'s at coarser spatial scales by aggregating inferences across multiple local areas. Specifically, given a region r consisting of a set of areas and a time period w, we estimate

(11) $$R_{r,w}=\frac{\sum_{i\in r,t\in w}\;R_{i,t}{\tilde{Z}}_{i,t}}{\sum_{i\in r,t\in w}\;{\tilde{Z}}_{i,t}}.$$

This definition is consistent with $R_{i,t}$ when r={i} and w={t}, and interprets $R_{r,w}$ as a summary statistic of the average number of secondary infections per primary infections over the region and time period.

**FIGURE 4:**
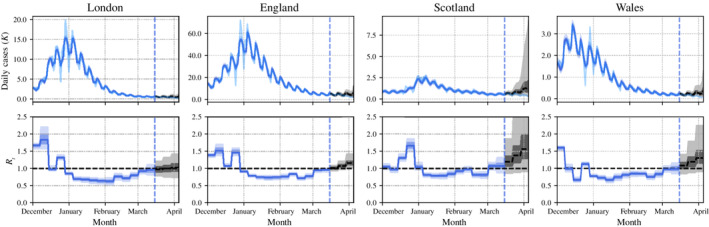
Regional estimates of cases and $R_{t}$ over the time period December 2020 to April 2021. Our model inferences are plotted in dark blue on both cases and $R_{t}$ plots, and additionally for case plots the true cases are plotted in light blue. Our model projections for cases and $R_{t}$, as well as 50% & 95% credible intervals, are plotted in grey. [Colour figure can be viewed at wileyonlinelibrary.com]

Figure 4 shows the posterior distributions of $R_{r,w}$, for the London NHS region, England, Scotland and Wales, and for each week in the December 2020 to March 2021 period, produced by the full EpiMap model with spatial length scale of 20 km, using data available on 15 March 2021. Corresponding plots for other English NHS regions can be found in Appendix C.3. Figure 4 shows sensible credible intervals both during the modelled 15‐week time period and subsequent 3‐week forecasts. In this example, we see that our model projects an increasingly uncertain size of epidemic in Scotland in the near future, with a non‐negligible probability of $R_{t}$ being above 1 in Scotland and Wales on 15 March 2021, whereas other regions are projected to have stable or shrinking epidemics.

## 5 DISCUSSION

We have proposed a hierarchical Bayesian approach to model epidemics at fine spatial scales, which incorporates movement of populations across local areas as well as spatiotemporal borrowing of strength. Empirical results suggest that our model can be a useful tool for policy‐makers to locate future epidemic hotspots early, in order to direct resources such as surge testing as well as targeted local transmission reduction measures.

As with other methods that infer the extent of epidemics through identified cases alone, the main limitations of this work are due to the provenance of the Pillars 1 + 2 case data. Firstly, there can be substantial selection bias in the population who get tested, leading to discrepancies between reported cases and the true size of the epidemic. In addition, the amount of testing may change over time, for example, due to localised testing or limited supplies of testing kits, potentially leading to spurious temporal patterns (Omori et al., 2020). Finally, case data are only reported for the combined Pillars 1 and 2 of the UK's testing regime. These correspond to different sectors of society at different points of an infection, with different delay distributions between infection and getting tested. Moreover, the proportion of tests under each pillar has been changing systematically since Pillar 2 testing began.

Our model is the result of a number of modelling choices, and can be improved in a number of ways. Firstly, our aim is to track local reproduction numbers and provide nowcasting of epidemic development in local areas, rather than understanding how NPIs affect transmission rates. This lead to our choice of a nonparametric GP prior for the reproduction numbers, rather than a generalised linear model relating transmission rates to NPIs. It is possible to extend our model to model effect of NPIs as in Flaxman et al. (2020). It also lead to our choice not to explicitly model the susceptible population, since it impacts the model just via lowered transmission rates.

Secondly, our model uses only Pillars 1 + 2 case data, which as noted above have biases that are not well understood. This affects our confidence in the inferred local transmission rates and forecasts. Further, in our model we assumed that positive test cases correspond 1‐1 to infections, which in fact does not hold due to asymptomatic infections. We can correct for these biases by incorporating less biased data like hospitalisation and death counts, as well as less granular but better understood estimates of prevalence data obtained from randomised surveys such as REACT (Riley et al., 2020) and the ONS infection survey (Pouwels et al., 2020).

In order to model cross‐area dependencies, we also used commuting flow data from the 2011 Census. However, this data does not necessarily reflect the commuter flow accurately during the pandemic, especially since the data is static. We used a simple approach to parameterise a time‐dependent flow matrix via $\alpha_{t}$ which captures the overall amount of travel in each week. Nonetheless, our model is likely to improve if this limitation is addressed by using more accurate, real‐time commuter flow data.

Finally, with the increasing importance of the roles of vaccines and variants, it is interesting to consider how these can be incorporated into our model. This will require a number of extensions, including separating the population into age bands and modelling the susceptible population. These extensions will incur significantly higher computational costs, and additional work will have to be performed with respect to software and implementational efficiency.

Our hierarchical Bayesian model is sensitive to a number of hyperparameters, particularly those specifying the generation interval and incubation period distributions, and the spatial and temporal length scales of the latent GP. These are hard to specify in a fully Bayesian manner. For example, the posterior strongly prefers spatial length scales that are too long due to model misspecification. Until there are good, fully Bayesian approaches to dealing with such situations, we have kept to a more pragmatic approach of using cut models and cross validation (see Section 3.1.2).

Our hierarchical model introduces stochasticity at all three layers of the model to capture different aspects of the unfolding epidemic. As a reviewer noted, there can be complex interplays between these layers, for example resulting in non‐identifiable parameters. The various components of the model have been chosen to avoid the worse of these, but we have not performed a systematic study of the impacts of these choices. This will be an illuminating piece of future research.

## Figures and Tables

**FIGURE C1:**
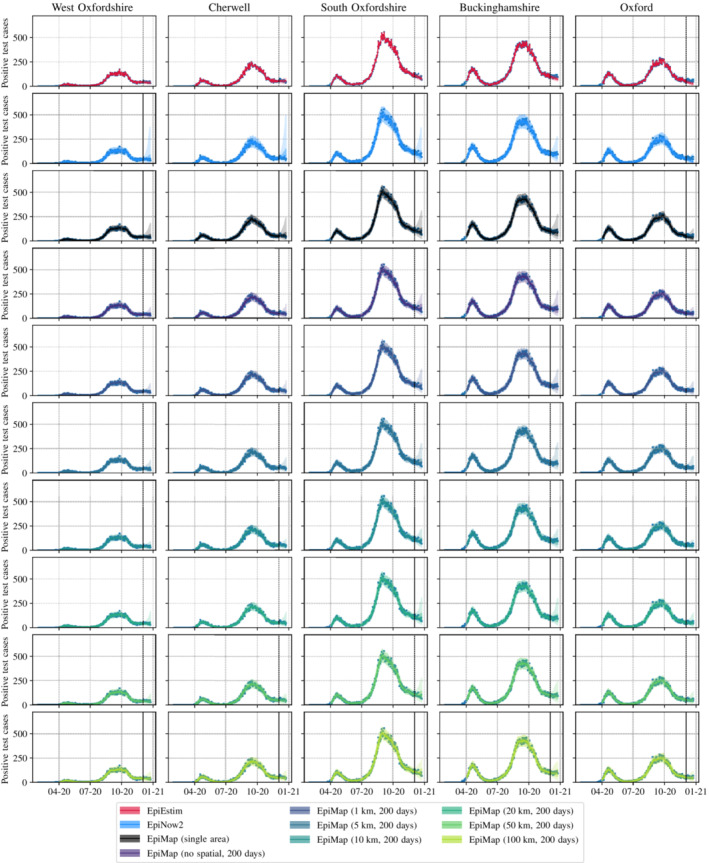
Estimated median, 50% (inner) and 95% (outer) credible intervals of the posterior predictive distributions for daily case counts plotted against the observed case counts used to infer $R_{t}$. Values to the left of the vertical line are inferred from data, and those to the right are future predictions. For EpiMap, we include the weekly variation in expected cases in the model, but plot the distribution without this variation for clarity. The EpiMap methods report the full distribution of expected cases. EpiNow2 returns the distribution of the mean number of cases. EpiEstim does not provide estimated case distributions, so future predictions are stochastic rollouts of the epidemic based on the last inferred $R_{t}$ distribution. [Colour figure can be viewed at wileyonlinelibrary.com]

**FIGURE C2:**
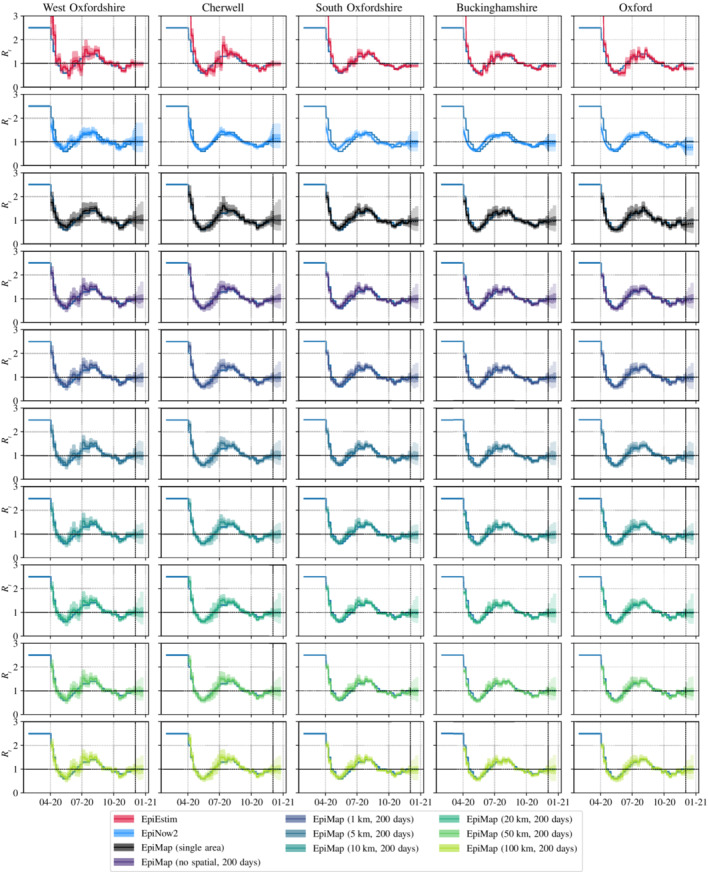
Estimated median, 50% (inner) and 95% (outer) credible intervals of $R_{t}$ for the methods plotted against the $R_{t}$ used to create the simulated epidemic. EpiEstim does not provide future estimates of $R_{t}$, and so the final $R_{t}$ posterior is used as a prediction. [Colour figure can be viewed at wileyonlinelibrary.com]

**FIGURE C3:**
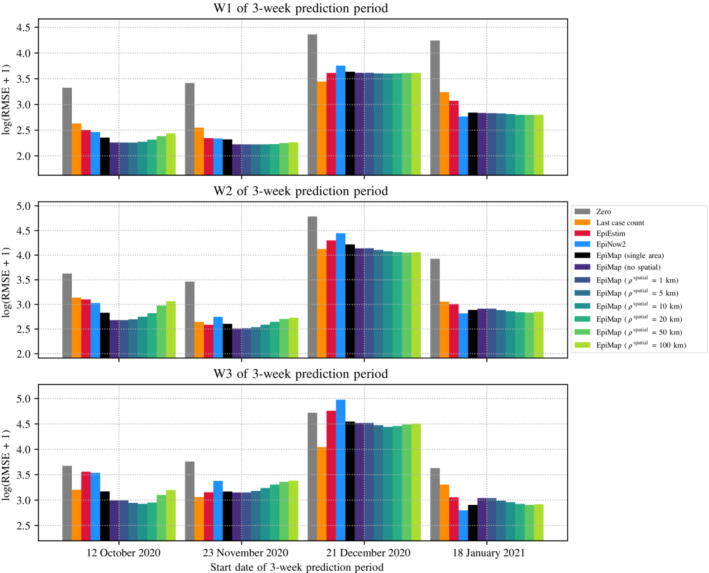
A comparison of predictive performance similar to Figure 2 but stratified by week over the 3‐week prediction period. As expected, the predictions made for later weeks, such as W3, are worse than those made for earlier weeks, such as W1, across models. The relative ordering of EpiMap variants typically remains unchanged for different weeks. [Colour figure can be viewed at wileyonlinelibrary.com]

**FIGURE C4:**
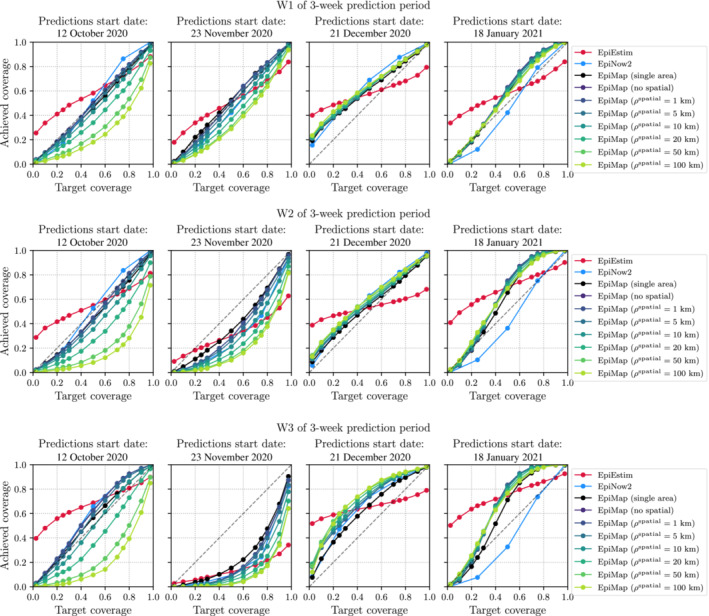
An evaluation of the uncertainty estimates produced by methods similar to Figure 2 but stratified by week over the 3‐week prediction period. As expected, the quality of uncertainty estimates degrades in the later weeks compared to earlier weeks, as indicated by reliability curves that are further from the ideal diagonal. Once again, the relative ordering of EpiMap variants typically remains unchanged for different weeks, however differences in uncertainty calibration between models tend to exacerbate. [Colour figure can be viewed at wileyonlinelibrary.com]

**FIGURE C5:**
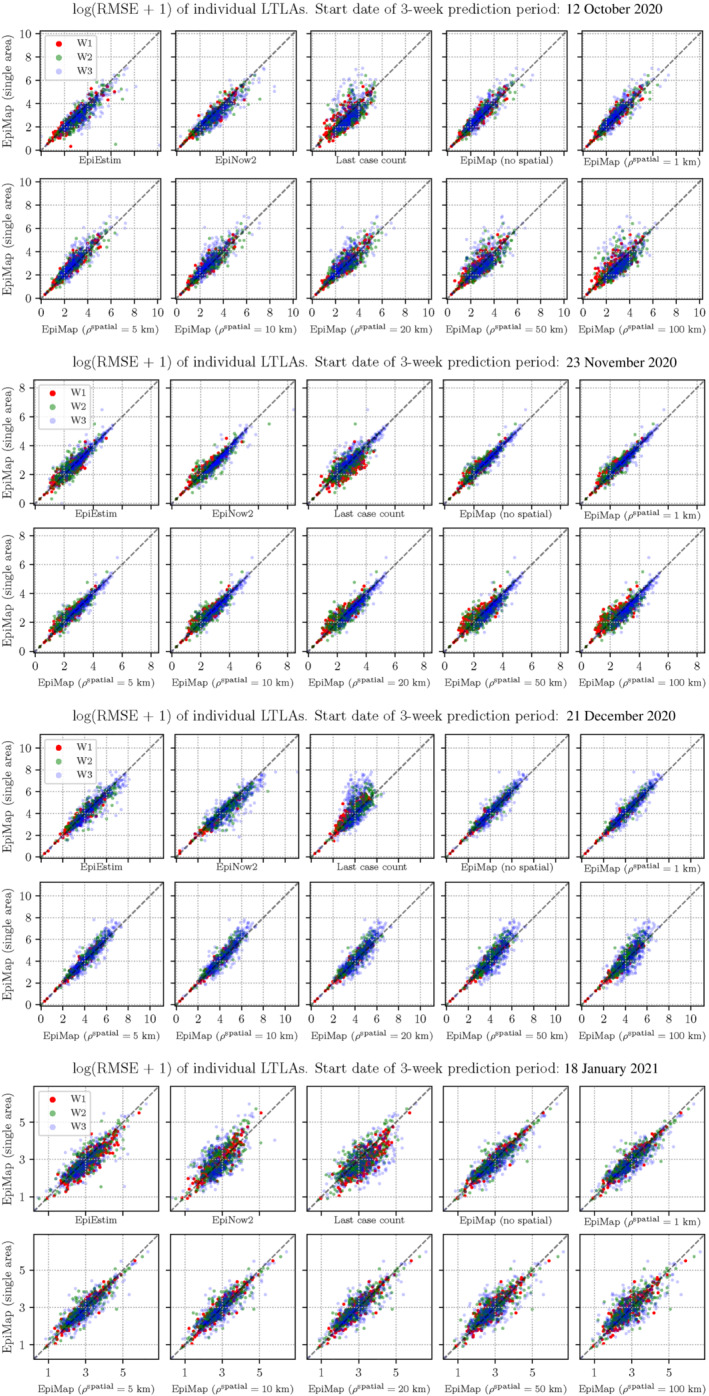
We plot the log(RMSE + 1) for individual lower‐tier local authorities (LTLAs; each dot is an LTLA) stratified by week. We observe variation in predictive performance for different LTLAs for all models, with large correlation in LTLA losses between methods. [Colour figure can be viewed at wileyonlinelibrary.com]

**FIGURE C6:**
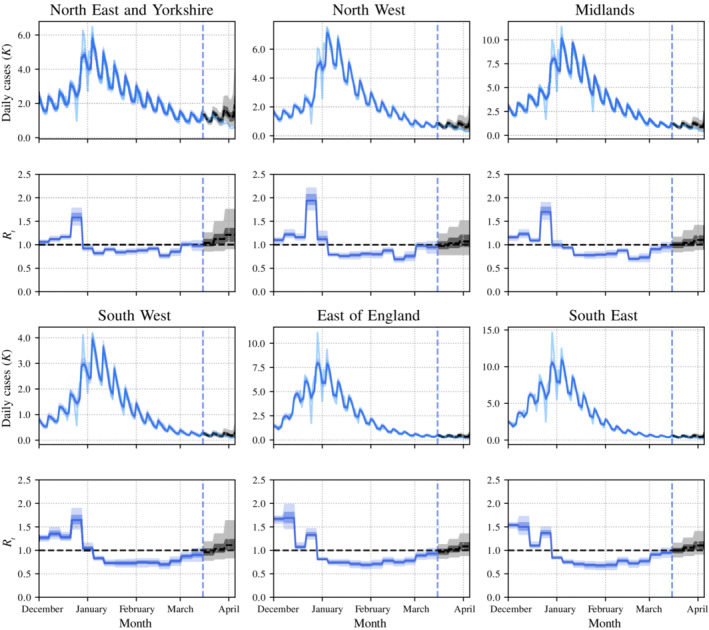
Additional regional estimates of cases and $R_{t}$ for NHS regions in England, using case data available by 15 March 2021. [Colour figure can be viewed at wileyonlinelibrary.com]

## Footnotes

1
https://www.gov.uk/government/publications/coronavirus‐covid‐19‐scaling‐up‐testing‐programmes

2
https://coronavirus.data.gov.uk

3
https://publichealthscotland.scot/our‐areas‐of‐work/sharing‐our‐data‐and‐intelligence/coronavirus‐covid‐19‐data‐and‐guidance/

4
https://phw.nhs.wales/topics/latest‐information‐on‐novel‐coronavirus‐covid‐19/

5
https://github.com/rs‐delve/covid19_datasets

6 We use ‘:’ to indicate the set of variables where the corresponding index ranges over all values.

7
https://census.ukdataservice.ac.uk/use‐data/guides/flow‐data.aspx

## APPENDIX A ADDITIONAL MODEL VARIATIONS

### A.1

In addition to the final model described in the main paper, we have also considered a number of model variations which did not result in improved performance so did not include them.

#### A.1 Global effects term in GP prior

We have also explored adding an additional global effects term to the spatial part of the kernel in (6):

(A1)
$$K_{ij}^{\text{space}}={\left(\sigma^{\text{spatial}}\right)}^{2}\exp(-\|y_{i}-y_{j}\|/\rho^{\text{spatial}})+{\left(\sigma^{\text{local}}\right)}^{2}\delta_{ij}+{\left(\sigma^{\text{global}}\right)}^{2}.$$

This has the effect of adding another GP term $f_{:}^{\text{global}}∼\text{GP}(0,\sigma^{\text{global}}K^{\text{time}})$ to (5) that is shared across all areas i=1,…,n. However this has an effect of over‐generalising estimates of $R_{i,t}$ from the high incidence areas (for which the likelihoods constrain inference of $R_{i,t}$ sufficiently) to the low incidence areas (for which they do not).

#### A.2 Modelling infectiousness and susceptibility separately

We have also explored a somewhat more elaborate meta‐population model. Note that in (3) and (4) the number of transmissions occurring in an area i depends only on $R_{i,t}$ and not on $R_{j,t}$ of the areas j that are ‘sending’ infections to area i. We can extend this to a model where the predicted mean count depends on properties of both the area that ‘receives’ an infection and the area that ‘sends’ it:

(A2)
$$C_{i,t}|C_{i,1:t-1}∼\text{NegBin}(\mu_{i,t},\psi )\;\mu_{i,t}=\overset{n}{\underset{j=1}{\sum }}\;R_{i,t}F_{ji}R_{j,t}^{\prime }Z_{j,t},$$

where $R_{j,t}^{\prime }$ can be interpreted as an infectiousness level of area j, and $R_{i,t}$ a susceptibility of area i, with the overall transmission rate being a function of both, as well as of the fluxes. While this extension is more complex and flexible, it is not clear whether both the infectiousness and susceptibilities are well‐identified from case count data. Empirically, we have not found it to perform differently from the simpler meta‐population model (3) and (4). We used the same GP prior for both the infectivities $R_{i,t}^{\prime }$ and susceptibilities $R_{i,t}$ in these experiments. As a result of the lack of statistical gains and of computational costs, we decided to use the simpler model (3)–(4).

## APPENDIX B COMPUTATIONAL EFFICIENCY CONSIDERATIONS

### B.1

#### B.1 Kronecker structured GP kernel

The Kronecker structure of the GP kernel allows for efficient computations (Flaxman et al., 2015; Saatçi, 2012). In particular, we never have to explicitly form or factorise $K^{\text{space}}⊗K^{\text{time}}$, which would have computational cost of $O({\left(nm\right)}^{3})$. Instead, if $f_{:,:}$ is represented as a n×m matrix, a draw from its GP prior can be expressed as:

(B1)
$$f_{:,:}=L^{\text{space}}E{\left(L^{\text{time}}\right)}^{⊤},$$

where $L^{\text{space}}$ and $L^{\text{time}}$ are Cholesky factors of $K^{\text{space}}$ and $K^{\text{time}}$, respectively, and E is an n×m matrix with iid standard normal distributed entries. The computational cost of this procedure is $O((n^{2}+m^{2})(n+m))$, which represents significant computational savings over $O({\left(nm\right)}^{3})$.

#### B.2 Positivised Gaussian approximation for infection model

We used a negative binomial distribution for the number of new infections on each day given infections in past days (4). This is a discrete distribution and makes posterior simulation, particular with the Stan probabilistic programming system, challenging. Instead we considered a simple approximation of the negative binomial distribution using a positivised Gaussian distribution with matched mean and variance. Specifically, if Y∼NegBin(μ,ϕ), we approximate $Y\approx |\tilde{Y}|$, where $\tilde{Y}∼𝒩(\mu ,\sqrt{(1+\phi )\mu })$. Note that $\left|\tilde{Y}\right|$ has mean higher than μ and variance lower than (1+ϕ)μ, but for μ≥5 the difference is practically negligible. However in cases where μ≤10 this can lead to under‐estimation of R, but we believe this is not a serious concern since the epidemic would then be of very small size anyway.

We chose this approximation as the computation for the infection model can be ‘reparameterised’ (Kingma & Welling, 2014) using the so‐called non‐centred parameterisation and lead to a better mixing MCMC sampler. Specifically, and assuming no meta‐population model for simplicity, we can write the sampling statements for the positivised Gaussian approximation of (4) as:

(B2)
$${\tilde{X}}_{i,t}=\left|R_{i,t}{\tilde{Z}}_{i,t}+\eta_{i,t}\sqrt{(1+\psi )R_{i,t}{\tilde{Z}}_{i,t}}\right|,\;\eta_{i,t}∼𝒩(0,1)\;\text{iid.}$$

Note that the modelled epidemic sizes ${\tilde{X}}_{i,:}$ can be written as a differentiable and efficiently computed function of a sequence of iid standard normal random variables $\eta_{i,:}$ (and the reproduction numbers). The gradients can be automatically computed by Stan, and the No‐U‐Turns Sampler mixes more effectively since while ${\tilde{X}}_{i,:}$ are highly correlated (which can lead to slow mixing if not reparameterised), the reparameterised random variables $\eta_{i,:}$ are independent a priori (hence faster mixing).

#### B.3 Regional inference

In order to track the daily evolution of the epidemic in real time it is preferable for the posterior simulations to run overnight. However, the model described in 3 is quite complex, and full posterior simulation for the whole of Great Britain using Markov chain Monte Carlo (MCMC) has significant computational costs. In this section we describe a two‐stage procedure to reduce the computational costs to a manageable level.

During the first stage the epidemic time courses of individual local areas are approximately inferred first by ignoring cross‐area dependencies in both the meta‐population infection model and the GP prior. This first stage can be easily parallelised across the 348 areas and completed quickly.

In the second stage, we split Great Britain into nine regions (seven NHS regions in England, plus Wales and Scotland), and modelled each region independently using the model described in Section 3. In order to account for transmissions to and from other regions, we fix the latent epidemic process for areas in other regions to the posterior median inferred during the first stage. To reduce the approximation error due to only modelling each region rather than the whole of Great Britain, we include in each region model a number of areas outside the region, such that for all areas within that region at least 80% of the off‐diagonal flux probabilities (corresponding rows in $F^{\text{fwd}}$ and $F^{\text{rev}}$) are included in the model.

## APPENDIX C ADDITIONAL FIGURES

### C.1

#### C.1 Simulation data

Figures C1 and C2 show case count and $R_{t}$ predictions for all models and variants of EpiMap.

#### C.2 Predicting future case counts

Figures C3 and C4 append the results in Figures 2 and 3, respectively, showing losses and uncertainty calibration stratified by week during the 3‐week prediction period. Figure C5 shows the log(RMSE + 1) for individual LTLAs which are stratified by week and compared between models.

#### C.3 Regional estimates

Figure C6 shows the regional estimates for cases and $R_{t}$ on remaining NHS regions in England, in the same setting as Figure 4.

## References

[rssa12971-bib-0001] Abbott, S. , Hellewell, J. , Thompson, R.N. , Sherratt, K. , Gibbs, H.P. , Bosse, N.I. et al. (2020) Estimating the time‐varying reproduction number of SARS‐CoV‐2 using national and subnational case counts. Wellcome Open Research, 5(112), 112.

[rssa12971-bib-0002] Bhatt, S. , Ferguson, N. , Flaxman, S. , Gandy, A. , Mishra, S. & Scott, J.A. (2020) Semi‐mechanistic Bayesian modeling of COVID‐19 with renewal processes. arXiv preprint arXiv:2012.00394.

[rssa12971-bib-0003] Bhoopchand, A. , Paleyes, A. , Donkers, K. , Tomasev, N. & Paquet, U. (2020) DELVE global COVID‐19 dataset . Available at: https://github.com/rs‐delve/covid19_datasets.

[rssa12971-bib-0004] Bi, Q. , Wu, Y. , Mei, S. , Ye, C. , Zou, X. , Zhang, Z. et al. (2020) Epidemiology and transmission of COVID‐19 in 391 cases and 1286 of their close contacts in Shenzhen, China: a retrospective cohort study. The Lancet Infectious Diseases, 20, 911–919. 32353347 10.1016/S1473-3099(20)30287-5 PMC7185944

[rssa12971-bib-0005] Carmona, C. & Nicholls, G. (2020) Semi‐modular inference: enhanced learning in multi‐modular models by tempering the influence of components. In: Chiappa, S. & Calandra, R. (Eds.) Proceedings of the 23rd International Conference on Artificial Intelligence and Statistics, volume 108 of Proceedings of Machine Learning Research. PMLR, pp. 4226–4235.

[rssa12971-bib-0006] Carpenter, B. , Gelman, A. , Hoffman, M.D. , Lee, D. , Goodrich, B. , Betancourt, M. et al. (2017) Stan: A probabilistic programming language. Journal of Statistical Software, 76(1), 1–32. 36568334 10.18637/jss.v076.i01 PMC9788645

[rssa12971-bib-0007] Cori, A. , Ferguson, N.M. , Fraser, C. & Cauchemez, S. (2013) A new framework and software to estimate time‐varying reproduction numbers during epidemics. American Journal of Epidemiology, 178(9), 1505–1512. 24043437 10.1093/aje/kwt133 PMC3816335

[rssa12971-bib-0008] Flaxman, S. , Mishra, S. , Gandy, A. , Unwin, H.J.T. , Mellan, T.A. , Coupland, H. et al. (2020) Estimating the effects of non‐pharmaceutical interventions on COVID‐19 in Europe. Nature, 584, 257–261. 32512579 10.1038/s41586-020-2405-7

[rssa12971-bib-0009] Flaxman, S. , Wilson, A. , Neill, D. , Nickisch, H. & Smola, A. (2015) Fast Kronecker inference in Gaussian processes with non‐Gaussian likelihoods. In: Proceedings of ICML. PMLR.

[rssa12971-bib-0010] Fraser, C. (2007) Estimating individual and household reproduction numbers in an emerging epidemic. PLoS One, 2(8), 1–12. 10.1371/journal.pone.0000758 PMC1950082 17712406

[rssa12971-bib-0011] Gostic, K.M. , McGough, L. , Baskerville, E.B. , Abbott, S. , Joshi, K. , Tedijanto, C . et al. (2020) Practical considerations for measuring the effective reproductive number, Rt. *medRxiv* . 10.1371/journal.pcbi.1008409 PMC7728287 33301457

[rssa12971-bib-0012] Hoffman, M.D. & Gelman, A. (2014) The no‐U‐turn sampler: adaptively setting path lengths in Hamiltonian Monte Carlo. Journal of Machine Learning Research, 15(47), 1593–1623.

[rssa12971-bib-0013] Jacob, P.E. , Murray, L.M. , Holmes, C.C. & Robert, C.P. (2017) Better together? Statistical learning in models made of modules .

[rssa12971-bib-0014] Kingma, D. & Welling, M. (2014) Auto‐encoding variational Bayes. In: ICLR 2014.

[rssa12971-bib-0015] Omori, R. , Mizumoto, K. & Chowell, G. (2020) Changes in testing rates could mask the novel Coronavirus disease (COVID‐19) growth rate. International Journal of Infectious Diseases, 94, 116–118. 32320809 10.1016/j.ijid.2020.04.021 PMC7167222

[rssa12971-bib-0016] Plummer, M. (2015) Cuts in Bayesian graphical models. Statistics and Computing, 25, 37–43.

[rssa12971-bib-0017] Pouwels, K.B. , House, T. , Pritchard, E. , Robotham, J.V. , Birrell, P.J. , Gelman, A. et al. (2020) Community prevalence of SARS‐CoV‐2 in England from April to November, 2020: results from the ONS coronavirus infection survey. The Lancet Public Health, 6, E30–E38. 33308423 10.1016/S2468-2667(20)30282-6 PMC7786000

[rssa12971-bib-0018] Riley, S. , Ainslie, K.E. , Eales, O. , Walters, C.E. , Wang, H. , Atchison, C . et al. (2020) Transient dynamics of SARS‐CoV‐2 as England exited national lockdown. *medRxiv* .

[rssa12971-bib-0019] Saatçi, Y . (2012) *Scalable Inference for Structured Gaussian Process Models*. PhD thesis, University of Cambridge.

[rssa12971-bib-0020] Wallinga, J. & Teunis, P. (2004) Different epidemic curves for severe acute respiratory syndrome reveal similar impacts of control measures. American Journal of Epidemiology, 160(6), 509–516. 15353409 10.1093/aje/kwh255 PMC7110200

